# Complete nucleotide sequence of a strain of cherry mottle leaf virus associated with peach wart disease in peach

**DOI:** 10.1007/s00705-013-1698-3

**Published:** 2013-05-07

**Authors:** Tefera A. Mekuria, Keri L. Druffel, James Susaimuthu, Kenneth C. Eastwell

**Affiliations:** 1 Department of Plant Pathology, Washington State University-I.A.R.E.C., 24106 North Bunn Road, Prosser, WA 99350 USA; 2 Department of Plant Pathology, Washington State University, P.O. Box 646430, Pullman, WA 99164-6430 USA; 3 Present Address: Department of Plant Pathology, University of Florida-GCREC, 14625 County Rd 672, Wimauma, FL 33598 USA

## Abstract

The complete nucleotide sequence and genome organization of a peach virus isolate from a naturally infected peach tree showing typical peach wart-like symptoms on the fruit surface was determined and compared to sequences of members of the family *Betaflexiviridae*. The genome consists of 7,987 nucleotides, excluding the poly-A tail, and has four open reading frames (ORFs). Analysis of the whole genome and putative proteins encoded by each ORF revealed greatest sequence similarity to a cherry isolate of cherry mottle leaf virus (CMLV). The two isolates have similar genome organizations and share 88 and 93 % homology in their corresponding products of the replicase and coat protein genes, respectively. CMLV has been reported from several *Prunus* spp. and may be associated with peach wart-like disease symptoms on peach fruit.

**Electronic supplementary material:**

The online version of this article (doi:10.1007/s00705-013-1698-3) contains supplementary material, which is available to authorized users.

Peach wart disease (PWD) is characterized by hard, wart-like outgrowths on the fruit surface [5]. Because PWD is graft transmissible, a viral agent has been assumed [5]. A peach tree (*Prunus persica* cv. Elberta) exhibiting peach wart symptoms (Fig. 1) was tested with a nested RT-PCR assay that detects members of the genera *Trichovirus*, *Foveavirus*, and *Capillovirus* [4]; the test yielded amplicons with sequences similar to that of cherry mottle leaf virus (CMLV, genus *Trichovirus,* family *Betaflexiviridae*). CMLV infects several species of *Prunus* and can be vectored by eriophyid mites, *Eriophyes inaequalis* [9]. RT-PCR assays of the PWD tree using virus-specific primers (Table S1) for other viruses known to infect peach yielded negative results.

**Fig. 1:**
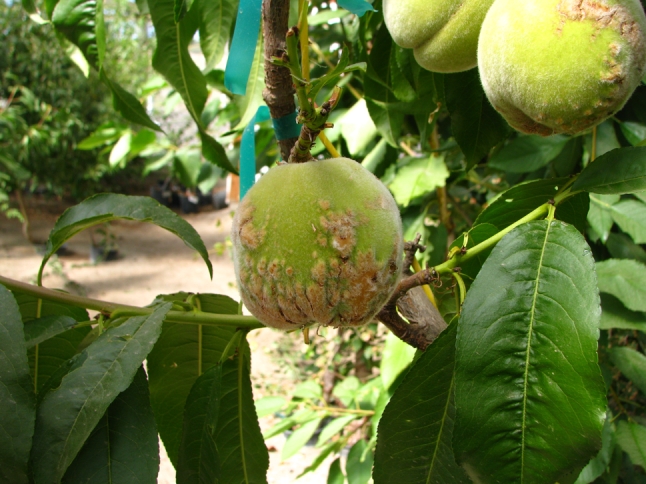
Peach tree (*Prunus persica* cv. Elberta) expressing peach wart disease (PWD) symptom on fruits. The tree is infected with cherry mottle leaf virus strain 95CI215 (GenBank KC207480)

To obtain additional sequence information, sequences from the 3′ region were amplified as described previously with an adapter primer [3] and CMLV-specific primers (Table S1). Products were ligated into pCR^®^ 2.1 (Invitrogen) and sequenced. Rapid amplification of cDNA ends was used to obtain the 5′ terminus of the RNA (5′-RACE; Invitrogen) with primers designed from the most upstream sequence (Table S1). Sequences were edited and assembled from at least eight overlapping fragments using CLC Main Workbench version 6 (CLC bio, Muehltal, Germany). Multiple sequence alignments were performed with Clustal W [11].

It is possible that a single agent or mixture of agents could cause PWD. In order to ascertain the presence of additional viruses in the original PWD-affected peach, total RNA from this source was subjected to deep sequencing. Multiplexed 50-bp single-end Illumina sequencing generated a total of 15,702,158 reads from this sample. The total *de novo* contigs generated were 26,187, with an average coverage per nucleotide of 1,186. Any contigs greater than 300 nt were compared to virus sequences in GenBank using the BLAST algorithm, and no signature sequences of viruses or virus-like agents other than CMLV were evident. A 7,998-nt fragment homologous to CMLV was assembled from 190,571 reads, and this virus sequence was 99 % identical to the sequence obtained from primer walking.

The complete genome of the virus isolate from the PWD-affected tree (designated strain 95CI215; GenBank KC207480) is 8,004 nt excluding the poly (A) tract and contains four putative open reading frames (ORFs). Its genome organization is typical of members of the genus *Trichovirus* (Fig. S1). Phylogenetic relationships to members of the family *Betaflexiviridae* were determined by the maximum-likelihood method in MEGA5 [10]. The PWD-associated strain 95CI215 (GenBank KC207480) co-segregated with CMLV from sweet cherry (strain SA1162-21; GenBank NC_002500)) (Fig. S2).

In pairwise comparisons between CMLV-SA1162-21 and the PWD-associated strain, the putative amino acid sequence identities of the four ORFs were 88, 83, 93 and 82 %, respectively, suggesting that the virus sequences represent the same virus species [1]. Hence, the virus from the PWD symptomatic tree is a strain of CMLV. However, ORF3, the putative coat protein coding region of cherry isolate of CMLV-SA1162-21, is 193 aa long [6], whereas ORF3 of the peach isolate CMLV-95CI215 is 260 aa long. To facilitate comparison of the amino acid sequences above, only 193 aa at the C-terminus of the CMLV-95CI215 coat protein sequence were used. Sequences of CMLV isolates SA1162-21 and 95CI215 possess upstream potential start codons at positions 6540 and 6539, respectively. However, the sequence of the cherry isolate is followed by two in-frame stop codons (Fig. S3) that are absent from the peach isolate. Moreover, the upstream start codon at position 6,539 of the peach isolate is in the context of Kozak consensus sequence requirements (A at position −3 and G at +4) for translation initiation [2, 8], which are not satisfied by the corresponding upstream start codon in the cherry isolate sequence or by the downstream start codons of either isolate. The configuration of ORF3 of the cherry isolate was confirmed by sequencing the corresponding region of CMLV cherry strain 8464-2/4 (GenBank KC241881) (Fig. S3). Thus, this sequence variation may represent host-specific adaptation of the virus genome.

The 5′UTR of CMLV-95CI215 shows highest similarity to that of peach mosaic virus (PcMoV) (83 %) rather than CMLV-SA1162-21 (80 %). CMLV is taxonomically and serologically related to PcMoV [7]. In contrast, the intergenic region between ORFs 3 and 4 is more closely related to that of CMLV-SA1162-21 (77 % identity) than to that of PcMoV (48 % identity). The 3′UTR of CMLV-95CI215 is 98 % identical to that of CMLV-SA1162-21, suggesting that conservation may be critical for virus replication [6].

The association of CMLV-95CI215 with PWD and the absence of additional virus signature sequences suggest that CMLV-95CI215 is the causal agent of PWD. The current study lays the groundwork for developing full-length infectious clones of the peach isolate to verify the role of CMLV-95CI215 in PWD of peach and to investigate the role ORF3 as a host range determinant.

## Electronic supplementary material

Below is the link to the electronic supplementary material.

Supplementary material 1 (DOC 132 kb)

Supplementary material 2 (DOC 361 kb)

Supplementary material 3 (DOC 52 kb)

Supplementary material 4 (DOC 123 kb)
