# Supplementary material for: Complete nucleotide sequence of a strain of cherry mottle leaf virus associated with peach wart disease in peach
Source: Arch Virol. 2013 May 7;158(10):2201–3. doi: 10.1007/s00705-013-1698-3 (PMC3785188; doi:10.1007/s00705-013-1698-3)
Supplement: Supplementary file 1 — Supplementary material 1 (DOC 132 kb) [file 705_2013_1698_MOESM1_ESM.doc]

**1k 2k 3k 4k 5k 6k 7k**

**MP**

**CP**


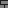


**NB**

**MP**

**CP**


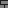


**NB**

**MP**

**CP**


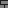


**MTR 2OFO Pep Hel RdRp**

**Replicase**

**MP**

**CP**


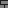


**NB**

**MTR 2OFO Pep Hel RdRp**

**CMLV-SA1162-21**

**(Cherry strain)**

**CMLV-95CI215**

**(Peach strain)**

**PcMoV**

**ACLSV**

**Fig. S1.** Genome organization of representative viruses in the genus *Trichovirus.* CMLV-95CI215: the PWD-associated isolate of *Cherry mottle leaf virus* (GenBank KC207480)*;* CMLV-SA1162-21: a cherry isolate of *Cherry mottle leaf virus* (GenBank NC_002500); PcMoV: *Peach mosaic virus*; ACLSV: *Apple chlorotic leaf spot virus*. Sequence motifs MTR: Viral methyltransferase; 2OFO= 2OG-Fe (II) oxygenase superfamily; Pep= UvrD/REP helicase; RdRp=RNA dependent RNA polymerase; CP=coat protein; NB=putative nucleic acid-binding protein. The CP of CMLV from peach strain is longer than CP of cherry strain.
