# Supplementary material for: Complete nucleotide sequence of a strain of cherry mottle leaf virus associated with peach wart disease in peach
Source: Arch Virol. 2013 May 7;158(10):2201–3. doi: 10.1007/s00705-013-1698-3 (PMC3785188; doi:10.1007/s00705-013-1698-3)
Supplement: Supplementary file 2 — Supplementary material 2 (DOC 361 kb) [file 705_2013_1698_MOESM2_ESM.doc]

⬟ *Hippeastrum latent virus*, NC_011540

⬟ *Hop mosaic virus*, NC_010538

⬟ *Hop mosaic virus*, NC_002552

⬟ *Cowpea mild mottle virus*, NC_014730

⬟ *Kalanchoe latent virus*, AFJ531635

⬟ *Blueberry scorch virus*, NC_003499

⬟ *Blueberry scorch virus*, AY941199

⬟ *Garlic latent virus*, NC_003557

⬟ *Red clover vein mosaic virus*, NC_012210

⬟ *Helleborus net necrosis virus*, NC_012038

⬟ *Poplar mosaic virus*, NC_005343

⬟ *Poplar mosaic virus*, X65102

🞽 *Grapevine rupestris stem pitting-associated virus*, FR691076

🞽 *Grapevine rupestris stem pitting-associated virus*, AF057136

🞽 *Peach chlorotic mottle virus*, NC_009892

🞽 *Apple stem pitting virus*, NC_003462

🞽 *Apple stem pitting virus*, EU095327

🞽 *Apple stem pitting virus*, FR694186

🞽 *Apricot latent virus*, NC_014821

🞽 *Apricot latent virus*, HQ339958

🞽 *Apricot latent virus*, HQ339957

🞽 *Apricot latent virus*, HQ339959

◖ *Cherry green ring mottle virus*, NC_001946

◖ *Cherry necrotic rusty mottle virus*, NC_002468

◖ *Cherry necrotic rusty mottle virus*, EU188438

◖ Cherry necrotic rusty mottle virus, EU188439

◖ *Banana mild mosaic virus*, AF314662

◖ *Potato virus T*, NC_011062

🞠 *Grapevine virus E*, NC_011106

🞠 *Grapevine virus A*, AF007415

🞠 *Grapevine virus B*, NC_003602

🟂 *Pear black necrotic leaf spot virus*, AY596172

🟂 *Citrus tatter leaf virus*, AY646511

🟂 *Apple stem grooving virus*, NC_001749

🟂 *Cherry virus A*, NC_003689

◖ *Fig latent virus* 1, FN377573

⯁ *Citrus leaf blotch virus*, EU857539

⯁ *Citrus leaf blotch virus*, EU857540

🞦 *Grapevine berry inner necrosis virus*, NC_015220

**🞦 *Cherry mottle leaf virus*, KC207480**

🞦 *Cherry mottle leaf virus*, NC_002500

🞦 *Peach mosaic virus*, DQ117579

🞦 *Apricot pseudo-chlorotic leaf spot virus*, NC_006946

🞦 *Apple chlorotic leaf spot virus*, EU223295

🞦 *Apple chlorotic leaf spot virus*, AB326225

🞦 *Apple spot chlorotic leaf spot trichovirus*, X99752

🞦 *Apple chlorotic leaf spot virus*, D14996

🞦 *Apple chlorotic leaf spot virus*, AB326223

*🞦 Apple chlorotic leaf spot virus*, AB326224

*🞦 Apple chlorotic leaf spot virus*, NC_001409

🞦 *Apple chlorotic leaf spot virus*, AJ243438

*Garlic virus E*, NC_004012

100

75

100

100

100

100

100

100

100

100

100

100

76

99

100

82

98

100

90

95

99

100

89

100

100

100

100

100

100

100

99

100

100

90

97

99

99

100

100

100

84

0.2

100

**Fig. S2.** Cladogram of the whole genome of selected members of the family *Betaflexiviridae* [Carla-(⬟), Capillo-(🟂), Citri-(⯁), Fovea-(🞽), Tricho-(🞦), Viti-(🞠) and unassigned-(◖) viruses]. The phylogenetic tree was generated using the Maximum Likelihood (ML) method in the MEGA5 analysis package. Branch significances were evaluated by constructing 1,000 trees in bootstrap analysis and the bootstrap values (≥65) are shown above the horizontal line at each node. *Garlic virus E* (GarVE) genome sequence is used as an out group.
