# Supplementary material for: Complete nucleotide sequence of a strain of cherry mottle leaf virus associated with peach wart disease in peach
Source: Arch Virol. 2013 May 7;158(10):2201–3. doi: 10.1007/s00705-013-1698-3 (PMC3785188; doi:10.1007/s00705-013-1698-3)
Supplement: Supplementary file 3 — Supplementary material 3 (DOC 52 kb) [file 705_2013_1698_MOESM3_ESM.doc]

CMLV-SA1162-21 ATGGATATGGGTCAAAAAGTGATCCAAGAAGGTCTAAAGGGAAAAGATTGGCCGGAGTTAAGTCGCTGGCCTTGGAAGGTGAATTGAATACATATTTAAACAAAGCTGGAGAAAGTAGTA

M D M G Q K V I Q E G L K G K D W P E L S R W P W K V N * I H I * T K L E K V V

CMLV-95CI215 ATGGATATGGATCGAAAAGTGACCCAAGAAGATCAAAAGGGAAAAGATTGGCCGCGGTTAAGTCACGACCTTTGGCAGGTGAATTTGATACTGAATACTTTAAAGCTGGAGAGAGTTGTG

M D M D R K V T Q E D Q K G K D W P R L S H D L W Q V N L I L N T L K L E R V V

8464-2/4 ATGGATATGGTTCGAAAAGTGATCCAAGAAGGTCAAAAGGGAAAAGATTGGCCGGGGTTAGGTCACTGGCGTTGGAGGGTGAATTGAATAAAAATTTTGGCAAAGCTGGAGAGGGTAGCT

M D M V R K V I Q E G Q K G K D W P G L G H W R W R V N * I K I L A K L E R V A

CMLV-SA1162-21 ACACAAGAGGAGTTGAAGCAACACATAGCAAGGTTCACAGGGGCAAAGGGAACCCTTTTGGAAAAAGTAAGGACTCACAGG**ATG**TCGGCGCGATTGAATCTAACGAACAAGATACATACC

T Q E E L K Q H I A R F T G A K G T L L E K V R T H R M S A R L N L T N K I H T

CMLV-95CI215 ACACAGAAGGAATTGAGGCAGCATATAAGGAGATTCACAGGAGCAAAGGGCACTCTTCTAGAAAAGATAAGGGCATACAGGATGTCGGCAAGACTCAATCTCACCAACAAAATTCAGACA

T Q K E L R Q H I R R F T G A K G T L L E K I R A Y R M S A R L N L T N K I Q T

8464-2/4 TCACAAGAGGATCTGAAAAGGCACATAGCGAGGTTTACAGGCACAAAAGGGACCCTACTAGAAAAGATAAGGACATACAGGATGTCGGCGAGATTGAATCTGACGAACAAAATACAGACA

S Q E D L K R H I A R F T G T K G T L L E K I R T Y R M S A R L N L T N K I Q T

**Fig. S3.** Comparison of starting position of open reading frame 3 in three *Cherry mottle leaf virus* (CMLV) isolates. Comparison was made at position 6540 to 6779 of reference sequence CMLV cherry isolate SA1162-21 (GenBank NC_002500) to peach isolate 95CI215 (GenBank KC207480) and cherry isolate 8464-2/4 (GenBank KC241881). The in-frame translation initiation positions for all the strains are shaded yellow. However, the red stars in boxed region show positions for early termination of translation in cherry strains.
