# Supplementary material for: Complete nucleotide sequence of a strain of cherry mottle leaf virus associated with peach wart disease in peach
Source: Arch Virol. 2013 May 7;158(10):2201–3. doi: 10.1007/s00705-013-1698-3 (PMC3785188; doi:10.1007/s00705-013-1698-3)
Supplement: Supplementary file 4 — Supplementary material 4 (DOC 123 kb) [file 705_2013_1698_MOESM4_ESM.doc]

**Table S1. Primers designed for RT-PCR amplification of the genome of *Cherry mottle leaf virus* from peach.**

| **Primer name** | **Primer sequence** | **Position relative**  **to genome** | **Orientation** | **Reference** |
| --- | --- | --- | --- | --- |
| **Cherry mottle leaf virus – peach isolate** | | | | |
| First strand primer | TATG­ACAC­GCGT­CGAC­TAGC­(T)_16_ | -- | antisense | [2] |
| AdPr | TATGACACGCGTCGACTAGC | -- | antisense | [2] |
| PW 5’RACE3 | GAAGGTTGCCAATTATCTC | 241 | antisense | This study |
| PW 5’RACE2 | CCTCTCTTCCTCTTTCTGCAACC | 273 | antisense | This study |
| PW 5’RACE1 | GGTAGAGCGTAAGAGAAGC | 302 | antisense | This study |
| CMLV V284 | CATTCTCATCCTGGTTGCAAAA | 367 | sense | This study |
| PW 708-133C | CCATTCGTTCCATTTTGC | 499 | antisense | This study |
| PW 708-221C | GATGGTTGGGTTAGATCTCC | 587 | antisense | This study |
| PWRACE_296R | TCCAGTTGAGGCCTTGACCAGTAA | 662 | antisense | This study |
| PWRACE_313R | TCAAATCGAGGAAGGTCTCC | 679 | antisense | This study |
| PWRACE_351R | AGGAAACACCATGGTAACCCAGAG | 717 | antisense | This study |
| PW 708F | GGTGAGAAGGTTTGGTCC | 987 | sense | This study |
| PW 709F | GCACTCAACCTTGATCTCGT | 2874 | sense | This study |
| PW 3550C | AAAGGTTCTGATGTCCATCT | 3495 | antisense | This study |
| PW 3600C | TCAAAGCACTTTCAAAGG | 3508 | antisense | This study |
| PWD3282F | GTTGCAAGCAAGCTACTTCAGCCA | 3648 | sense | This study |
| PW 607F | TTGGTGTGGGAGTTATCG | 3980 | sense | This study |
| PWD3880R | ATCTCTCTTCCCTATCCATCTC | 4267 | antisense | This study |
| PW 608C | GAATCAATGTAGGCAGGAAT | 4731 | sense | This study |
| 4940C | GGACTAAATTCAACGAGGAT | 4930 | sense | This study |
| PW-F1 | GTTGAATTTAGTCCATGGTGCAG | 4936 | sense | This study |
| PW-F1 | GTTGAATTTAGTCCATGGTGCAG | 4937 | sense | This study |
| PW5150 | CTGAGGTATGTAGGATGGG | 4949 | sense | This study |
| 4940C: | GGACTAAATTCAACGAGGAT | 4949 | antisense | This study |
| 4980C | GCAGAAAGGATTTTTTCAAC | 4982 | sense | This study |
| 4980C: | GCAGAAAGGATTTTTTCAAC | 4982 | antisense | This study |
| 5150 | CTGAGGTATGTAGGATGGG | 5143 | sense | This study |
| PW-F2 | GGTTGAACTCCTGAGGTATGTA | 5154 | sense | This study |
| PW 655C | CCTTCAGGCTCAGCTTTT | 5419 | antisense | This study |
| CMLV V5775 | ACAAGTCAAAAATCGCAGAA | 5757 | sense | This study |
| PW 657F | CAAAAGGGCCTCTGATCT | 5836 | sense | This study |
| PW 676F | TCAGTTTCTCTCGATGACCC | 6170 | sense | This study |
| PW 675F | AGCTCCAGCTGAATCTATGT | 6361 | antisense | This study |
| PWD6055F | TTAGCTTTGCTGAGGCTGTACCGA | 6420 | sense | This study |
| PW 656F | TCTCCTGTTCTGATCGCT | 6822 | antisense | This study |
| PWD6485R | ACGTCCCTTGGATTGCAATGTTGG | 6873 | antisense | This study |
| CMLV 7030C | TCATCCTCGTTTGTTGACGC | 7003 | antisense | This study |
| **TriFoCap degenerate primers for Trichoviruses, Foveaviruses and Capillovirsues** | | | | |
| PDO-F1i | TITTYATKAARWSICARYWITGIAC |  |  | [4] |
| PDO-R3i | GCRCACATRTCRTCICCIGCRAAIIA |  |  |
| PDO-R4i | ARIYICCATCCRCARAAMITIGG |  |  |
| **Nepovirus degenerate primer for Tomato ringspot-, Peach rosette mosaic-, Strawberry latent ringspot-, Tobacco ringspot- and Peach enation viruses** | | | | |
| NEPOR1 | WVDKDRYNWATGGWGATG |  | sense | [3] |
| AdPr | TATGACACGCGTCGACTAGC |  | antisense |
| **Plum bark necrosis stem pitting associated virus** | | | | |
| PBNSPaV det F | TACCGAAGAGGGTTTGGATG |  | sense | [1] |
| PBNSPaV det R | AGTCGCACCACCAGTCTTCT |  | antisense |
| **Prunus necrotic ring spot virus** | | | | |
| PNRSV 16 | ATATTGGCAGGTACAGAAGG |  | sense | [7] |
| PNRSV 17 | TTCGGAGAAATTCGAGTGTGC |  | antisense |  |
| **Apple mosaic virus** | | | | |
| OPS 6 | TTCAACATGGTCTGCAAGT |  | sense | Patrick J. Shiel, personal communication |
| OPS 13 | GCCTCCTAATCGGGGCATCA |  | antisense |
| **Prune dwarf virus** | | | | |
| PDV 3 | CCCTCCTGCTGGTTTTGTTA |  | sense | [6] |
| PDV 5 | CACGGACTTTCATGGTGTAA |  | antisense |
| **Hop stunt viroid** | | | | |
| HSVd1 | GCCCCGGGGCTCCTTTCTCAGGTAAG |  | sense | Modified from [5] |
| HSVd1 | GGCAACTCTTCTCAGAATCC |  | antisense |

1. Al Rwahnih M, Uyemoto JK, Falk BW, Rowhani A (2007) Molecular characterization and detection of plum bark necrosis stem pitting-associated virus. Arch Virol. 152:2197-206
2. Eastwell KC, du Toit LJ, Druffel KL (2009) Helleborus net necrosis virus: A new *Carlavirus* associated with ‘black death’ of *Helleborus* spp. Plant Dis. 93:332-338
3. Eastwell KC, Mekuria TA, Druffel KL (2012) Complete nucleotide sequences and genome organization of a cherry isolate of cherry leaf roll virus. Arch Virol 157:761–764
4. Foissac X, Svanella-Dumas L, Gentit P, Dulucq MJ, Marais A, Candresse T (2005) Polyvalent degenerate oligonucleotides reverse transcription-polymerase chain reaction: a polyvalent detection and characterization tool for *Trichoviruses*, *Capilloviruses* and *Foveaviruses*. Phytopathology 95:617–625
5. Kusano N, Shimomura K (1997) Selection of PCR primers and a simple extraction method for detection of *Hop stunt viroid*-plum in plum by Reverse transcription polymerase chain reaction. Ann Phytopathol Soc Jpn 63: 119-123
6. Rampitsch C, Eastwell KC, Hall J (1995) Setting confidence limits for the detection of prune dwarf virus in Prunus avium with a monoclonal antibody-based triple antibody-sandwich ELISA. Ann appl Biol. 126: 485-491
7. Vaskova D, Petrzik K, Karesova R, (2000) Variability and molecular typing of the woody-tree infecting Prunus necrotic ringspot ilarvirus. Arch Virol 145: 699-709
